# Climate change models predict southerly shift of the cat flea (*Ctenocephalides felis*) distribution in Australia

**DOI:** 10.1186/s13071-019-3399-6

**Published:** 2019-03-22

**Authors:** Nicole Crkvencic, Jan Šlapeta

**Affiliations:** Sydney School of Veterinary Science, Faculty of Science, University of Sydney, Sydney, New South Wales 2006 Australia

**Keywords:** Bioclimatic variables, *cox*1, Haplotypes, Maxent

## Abstract

**Background:**

Bioclimatic variables play an integral part in the life-cycle of *Ctenocephalides felis*, the most common flea found on companion animals. It is essential that we understand the effects of climate on *C. felis* distribution as fleas are a major veterinary and public health concern. This study investigated the current distribution of *C. felis* in Australia and future projections based on climate modelling.

**Results:**

Typing of *C. felis* was undertaken using the cytochrome *c* oxidase subunit 1 (*cox*1) mitochondrial DNA (mtDNA) region and current distribution of haplotypes was mapped by Maximum Entropy (Maxent) niche modelling. All *C. felis* haplotypes have been predicted to persist in environments along the eastern and southern coastlines of Australia and distinct ecological niches were observed for two *C. felis* haplogroups. Clade ‘Cairns’ haplogroup thrives under the northern coastal tropical conditions whilst Clade ‘Sydney’ haplogroup persists in temperate climates along the eastern and southern coasts. The model was then used to predict areas that are projected to have suitable climatic conditions for these haplogroups in 2050 and 2070 under the Intergovernmental Panel on Climate Change (IPCC) climate change scenarios. Under all IPCC Representative Concentration Pathways (RCP) climate change scenarios, the geographical range of all haplotypes was reduced by 5.59–42.21% in 2050 and 27.08–58.82% by 2070. The ranges of all clades were predicted to shift south along the eastern coastline.

**Conclusions:**

As future temperatures exceed critical threshold temperatures for *C. felis* development in the northern tropical areas, Clade ‘Cairns’ haplogroup is predicted to shift south along the coastline and possibly outcompete the temperate haplogroup in these areas. If *C. felis* haplogroups possess distinct climatic niches it suggests a potential for these to be biologically distinct and have differing developmental rates and vector capabilities.

**Electronic supplementary material:**

The online version of this article (10.1186/s13071-019-3399-6) contains supplementary material, which is available to authorized users.

## Background

*Ctenocephalides felis* (Siphonaptera: Pulicidae), commonly known as the cat flea, is the most common flea found on companion animals in Australia [1, 2]. *Ctenocephalides felis* has a cosmopolitan distribution and is highly tolerant to a wide range of environmental conditions [3]. As *C. felis* imposes health risks to humans and domestic animals as a biological vector, it is important to understand and predict both current and future suitable habitats [3]. Climatic variables play an integral part in the life-cycle of *C. felis* and consequently have an effect on their distribution, as they will only reside and successfully reproduce within limited climatic ranges [4]. As a result, it is essential that we understand the effects of climate on *C. felis* distribution, both now and in the future.

Recent studies have investigated the molecular diversity of *C. felis* in Australia at the cytochrome *c* oxidase subunit 1 (*cox*1) and cytochrome *c* oxidase subunit 2 (*cox*2) mitochondrial DNA (mtDNA) regions [1, 2, 5–7]. Two genetically distinct subpopulations were identified, with one population located along the eastern and southern coasts and the other strictly located in the northern city of Cairns, Australia. It is currently unknown what biological factors are governing these distributions and whether the Cairns subpopulation is restricted to this area.

Climate change is anticipated to alter the ranges of zoonotic parasite species and consequently, associated disease emergence within human and domestic animal populations in the future [4, 8]. The effect of climate change can have the potential to shift tropical populations into temperate areas as bioclimatic norms exceed critical threshold temperatures for parasite survival [9]. In a predictive model study, the distribution of *C. felis* in Spain was predicted to expand to newly suitable habitats as a result of climate change [10]. This could be the circumstance in Australia, where the distribution of *C. felis* subpopulations may shift to newly suitable habitats. Ecological niche modelling such as the Maximum Entropy (Maxent) model [8, 11] in epidemiology is a useful tool as it can assess the relative importance of bioclimatic variables and use these factors to predict changes in the distribution of parasites and their pathogens over time [12].

The aim of this study is to model if *C. felis* distribution in Australia will be affected by the Intergovernmental Panel on Climate Change (IPCC) climate change scenarios. To address this aim, we evaluated the genetic diversity of the cat flea by polymerase chain reaction (PCR) amplification and sequencing of the *cox*1 mtDNA region. The predictive Maxent modelling was then used to determine the current and future distribution of *C. felis* haplotypes in Australia using IPCC climate change scenarios.

## Methods

### Flea specimens

Fleas were collected opportunistically from domestic cats and dogs presented to various veterinary clinics across the north-eastern region of Queensland (Additional file 1: Table S1). Fleas from individual animals were collected by veterinarians in 1.5 ml Eppendorf tubes, labelled with the postcode of the veterinary clinic, and stored in 80–100% ethanol at -20 °C for transport to The University of Sydney Veterinary Parasitology Diagnostic Laboratory. In addition, 65 *C. felis* samples from previous studies that have been published on GenBank and 33 unpublished *C. felis* genotyped samples were included (Additional file 1: Table S2) [1, 2, 5]. These additional samples were collected from veterinary clinics in all seven states and territories across Australia and were used to increase the sensitivity of the ecological niche model. There is a limitation in the extent of flea locality in this study as samples were collected from dogs or cats presented at veterinary clinics within their local area where these animals have acquired these fleas from an unknown locality.

### Morphological identification of flea specimens

Fleas were identified morphologically using a stereo microscope (5–20× objective) with the aid of identification keys and descriptions [13, 14].

### Extraction of total DNA and mounting of flea exoskeletons

Total flea DNA was extracted using an ISOLATE II Genomic DNA kit (Bioline, Eveleigh, Australia) according to manufacturer’s protocol with a few modifications as previously described [2]. Briefly, an incision was made on the anterior dorsal section of the flea abdomen using a sterile scalpel blade and digested overnight with 25 µl Proteinase K and 180 µl of lysis buffer at 56 °C. The remaining steps were as per manufacturers protocols. The total DNA was eluted into 50 µl of elution buffer and stored at -20 °C until analysis by PCR. Exoskeletons were placed in 10% potassium hydroxide for 12 h, rinsed with RO water and dehydrated in an ethanol series (70%, 80%, 95% and 100%) for 1 h each. The specimens were then slide-mounted in Euparal (Australian Entomological Supplies, Coorabell Australia).

### Amplification of the *cox*1 gene by PCR and sequence analysis

The 601-nt fragment of the 5′-end of the *cox*1 gene sequence from the mtDNA region was amplified by PCR using a generic invertebrate amplification forward primer, LCO1490 [S0867] (5′-GGT CAA CAA ATC ATA AAG ATA TTG G-3′) and a previously-designed reverse primer, Cff-R [S0368] (5′-GAA GGG TCA AAG AAT GAT GT-3′) [2, 15]. Each reaction was conducted at a final volume of 30 µl, containing 15 µl MyTaq™ Red Mix (Bioline, Eveleigh, Australia) and approximately 2 µl of genomic DNA template. The PCR was conducted using C100 Thermal Cycler (BioRad, Gladesville, Australia) with cycling parameters set as following: denaturing at 95 °C for 1 min followed by 35 cycles at 95 °C for 15 s, 55 °C for 15 s and 72 °C for 10 s, followed by a final elongation for 5 min at 72 °C. All reactions were run with a negative control using sterile PCR-grade water [1, 2]. Resulting amplicons were visualised on a 1% (w/v) agarose gel with GelRed™ Nucleic Acid Gel Stain (Biotium, Fremont, CA, USA) in 1× TBE buffer to verify product size. Samples found to yield an unambiguous single band of expected size were sent for bidirectional sequencing (Macrogen Ltd, Seoul, Korea). Individual sequences were assembled and aligned against a reference sequence previously submitted to GenBank (KF684884) [2] using CLC Main Workbench 6.9.1 (CLC bio, Qiagen, Aarhus, Denmark).

### Modelling of current *C. felis* distribution in Australia using Maxent

A Maxent model [8, 11] was used to model the current distribution of *C. felis* in Australia. A total of 179 samples were used, which included samples obtained in this study, unpublished data and published data (Additional file 2: Table S4). Latitude and longitude coordinates for each sample were entered into Biodiversity and Climate Change Virtual Laboratory (BCCVL) (BCCVL, Griffith University) as biological data [16]. “Australia, Current Climate (1976–2005), 30 arcsec (~1 km)” climate and environmental data was selected from the datasets available in the BCCVL collections. All variables were initially included in the model, while only the variables with the highest probability of *C. felis* presence in response to ecogeographical variables were included in the final model as they reported the greatest amount of the observed variation. The chosen response variables were required to be within the environmental thresholds for *C. felis* survival. Namely, temperatures between 3–35 °C with 70–95% humidity and high precipitation levels (> 500 mm yearly) [17, 18]. Receiver operating characteristics (ROC) curves were assessed, where the area under the curve (AUC) was used to evaluate the accuracy of the resulting model. A model with an AUC score of 0.5 or below indicated that the model performed no better than random. Models with an AUC score of 1 indicated a perfect model. Additionally, the variable importance function was used to calculate the correlation score between the standard prediction and the new prediction, giving an estimation (%) of the importance of that variable in the model. The higher the value of the variable, the more influential it was in the model. The correlation matrix was used to complement the variable importance function where it showed the probability of predictor variables to be correlated to ensure that the included variables are not highly correlated and produce a bias output in the model. It is important to note that there are limitations involved in strictly using AUC models, and they should be used in conjunction with other model evaluation methods [19]. Therefore, we used the variable importance function and correlation matrix with the AUC scores to determine the model of best-fit for the current distribution of *C. felis*.

### Maxent modelling of predicted future distribution of *C. felis* in Australia under IPCC climate change models

The species distribution models were analysed further using a Maxent climate change model in BCCVL for predicted future distribution of *C. felis* in Australia. Four IPCC Representative Concentration Pathways (RCP) (2.6, 4.5, 6.0 and 8.5) for the 2050s and 2070s were evaluated: WorldClim, future projection using IPSL-CM5A-LR RCP2.6 (IMAGE [20]), 10 arcmin (2050), WorldClim, future projection using IPSL-CM5A-LR RCP4.5 (CGAM [21]), 10 arcmin (2050), WorldClim, future projection using IPSL-CM5A-LR RCP6.0 (AIM [22]), 10 arcmin (2050), WorldClim, future projection using IPSL-CM5A-LR RCP8.5 (MESSAGE [23]), 10 arcmin (2050), WorldClim, future projection using IPSL-CM5A-LR RCP2.6, 10 arcmin (2070), WorldClim, future projection using IPSL-CM5A-LR RCP4.5, 10 arcmin (2070), WorldClim, future projection using IPSL-CM5A-LR RCP6.0, 10 arcmin (2070), WorldClim, future projection using IPSL-CM5A-LR RCP8.5, 10 arcmin (2070). The change in area was calculated by examining centre of range, contraction and expansion of the range.

## Results

### Three divergent *cox*1 haplogroups of *C. felis* are present throughout the north-eastern region of Australia

**Fig. 1:**
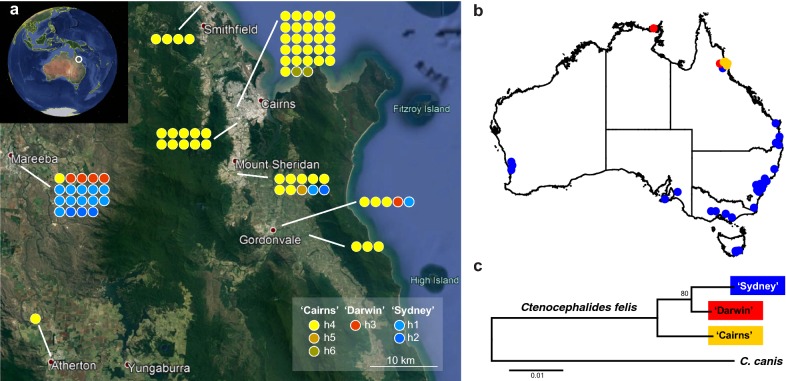
Map of locations of *Ctenocephalides felis* collections sites in Australia. **a** Locations of *C. felis* collections sites (*n* = 179) used in this study. Coloured circles represent locations and are colour-coded according to the *C. felis* Clade. **b** Summary of locations in North Eastern Australia with distribution of all three clades of *C. felis* obtained new in this study (DigiGlobe, 2018 and Google 2018; Data SIO, NOAA, U.S. Navy, NGA, GEBCO). The *C. felis* clades and haplotypes are colour-coded and each circle represents individual flea specimen. Inset: location of the area on the map of Australia (Image Landsat/Copernicus, Google 2018, US Dept of State Geographer; Data SIO, NOAA, U.S. Navy, NGA, GEBCO). **c** Phylogenetic relationships based on 601 nt of *cox*1 of the three *C. felis* clades reconstructed using Minimum Evolution with K2P distance matrix and bootstrapped (1000 replicates) and rooted with *Ctenocephalides canis*

Cat fleas, *C. felis*, were genotyped using the mtDNA 5'-end of the *cox*1 gene (601 bp). Genotyping of *C. felis* (*n* = 81) collected from nine locations in north-eastern region of Australia revealed six *cox*1 haplotypes (h1–6; Fig. 1, Additional file 1: Table S3). The majority of the fleas were either haplotype h1 (*n* = 14, 17.28%) or h4 (*n* = 55, 67.90%). Pairwise comparison between haplotypes h1 and h4 revealed 97.18% identity (15 nt differences). The six haplotypes clustered into three different clades: Clade ‘Sydney’, Clade ‘Darwin’ and Clade ‘Cairns’. Clade ‘Sydney’ forms a haplogroup of haplotypes h1 (*n* = 14) and h2 (*n* = 4) that differ by a single synonymous nucleotide polymorphism (SNP). Clade ‘Darwin’ is formed from a single haplotype, h3 (*n* = 5), and is divergent from Clade ‘Sydney’ with nucleotide variations at 6 and 5 of 601 positions when compared to haplotypes h1 and h2, respectively. Clade ‘Cairns’ forms a haplogroup of haplotypes h4 (*n* = 55), h5 (*n* = 1) and h6 (*n* = 2) that differ by a SNP and is divergent from Clade ‘Sydney’ and Clade ‘Darwin’. The *cox*1 amino acid sequences of all clades are 100% identical.

### Species distribution modelling confirms a geographical niche for Clade ‘Cairns’ and the nation-wide distribution of Clade ‘Sydney’

**Fig. 2:**
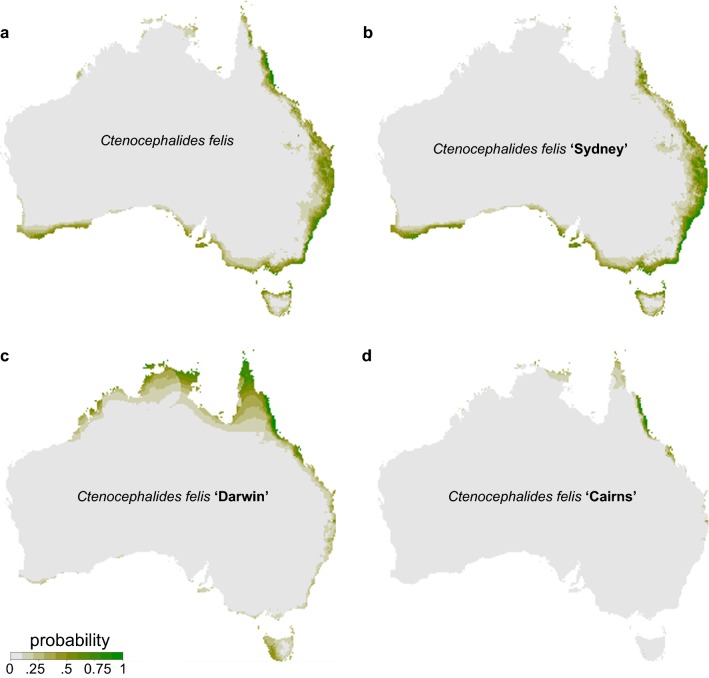
The current distribution of all *Ctenocephalides felis* haplotypes in Australia. Predictions using the best-fit Maxent model for *Ctenocephalides felis* (**a**), Clade ‘Sydney’ (**b**), Clade ‘Darwin’ (**c**) and Clade ‘Cairns’ (**d**) are shown on the maps. An ecological niche in the tropical region of Australia has been suggested for Clade ‘Cairns’, whereas Clade ‘Sydney’ is found nation-wide. A total of 179 samples were used as biological data with Australia’s current climate (1976–2005) and 30 arcsec resolution. Environmental variables met the thresholds for *C. felis* survival, including temperatures between 3–35 °C with 70–95% humidity and high precipitation levels (> 500 mm yearly)

An additional 98 samples were used in conjunction with the 81 samples obtained from this study to model the species distribution of *C. felis* in Australia (*n* = 179; Fig. 1). The best Maxent model included a fine scale with the following four environmental variables: maximum temperature of warmest month, mean temperature of coldest quarter, precipitation of wettest quarter and precipitation of warmest quarter (Additional file 3: Figure S1). The best model was determined by interpreting AUC values, variable importance functions and the correlation matrix (Fig. 2). The AUC for Clade ‘Sydney’, Clade ‘Darwin’, Clade ‘Cairns’ and all haplotypes were 0.96, 0.99, 1.0 and 0.98, respectively. The variable importance function identified that the maximum temperature of warmest month, mean temperature of coldest quarter, precipitation of wettest quarter and precipitation of warmest quarter variables had an importance of 10%, 40%, 25% and 25%, respectively, in the model. The correlation matrix showed that precipitation of wettest quarter and precipitation of warmest quarter are highly correlated (0.5) whereas all other variables are uncorrelated with each other.

Areas that had a suitability > 50% for Clade ‘Sydney’, Clade ‘Darwin’ and Clade ‘Cairns’ had a maximum temperature of 35 °C, 40 °C and 35 °C, respectively, in the warmest month, mean temperature of 10 °C, 15 °C and 25 °C, respectively, in the coldest quarter, precipitation of 250 mm, 1700 mm and 500 mm, respectively, during the wettest quarter and lastly, a precipitation of 1400 mm, 1400 mm and 1500 mm, respectively, during the warmest quarter (Fig. 2). These conditions fall within the required thresholds for survival of *C. felis.* Clade ‘Sydney’ was observed to be found along the entirety of east coast of Australia, extending to the coast of South Australia, Tasmania and Perth (Fig. 2). Areas with suitability for the Clade ‘Darwin’ were primarily found along the northern coastline of Australia, the north to middle parts of the east coast and the western coast of Tasmania (Fig. 2). In contrast, suitable habitats for Clade ‘Cairns’ were primarily found in the northern and north-eastern tropical areas of Australia, in particular Townsville, Cairns and the surrounding region (Fig. 2).

### Modelling of potential future distribution predicts the range of *C. felis* in Australia to shift south along the eastern coastal regions

**Fig. 3:**
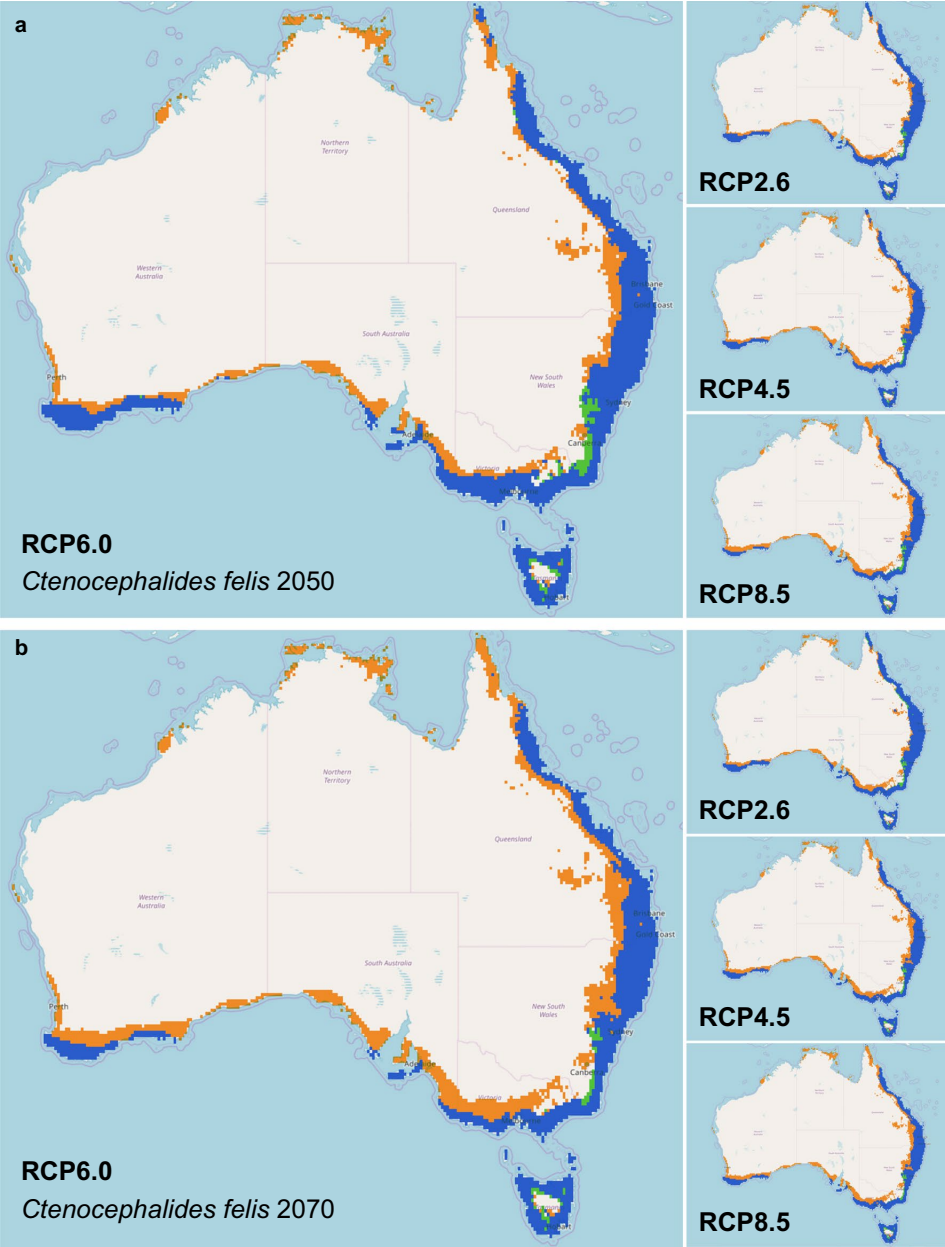
Comparison of climate change models of all *Ctenocephalides felis* haplotypes for 2050 and 2070 in Australia. In 2050 (**a**) and 2070 (**b**), the distribution of *C. felis* in Australia is predicted to shift south along the east coast. The range is predicted to be restricted even closer to the coast as the inner regions of Australia become too warm and dry for flea survival. Orange areas indicate a contraction from the current range, blue areas indicate no change in range and green areas indicate an expansion of the current range. The species distribution models were analysed further using a Maxent climate change model for predicted future distribution of *C. felis* in Australia. IPCC Representative Concentration Pathways (RCP) (2.6, 4.5, 6.0, and 8.5) for the 2050 (**a**) and 2070 (**b**) were evaluated with WorldClim data and 10 arcmin resolution

Areas with suitable climatic conditions for *C. felis* in Australia have been predicted to decrease by 2050 and even further by 2070 under all four RCP scenarios (2.6, 4.5, 6.0 and 8.5; Table 1). Currently, 959,040.82 km^2^ is suitable for *C. felis* in Australia. However, in 2050 the suitable area reduced in all four scenarios, ranging between 548,751.36–680,130.58 km^2^, leaving 54.45–68.61% of the area in common with the current model (Table 1, Fig. 3). By the 2070s, the area of suitability declined even further in all four models with 386,233.19–717,111.65 km^2^ suitable for *C. felis*, leaving 27.82–70.26% of the area in common with the current model (Table 1, Fig. 3). The contracted areas are found in the current northern parts of the *C. felis* range where the geometric centre of the species range shifted 397.14 km south along the east coast by the 2050s (Table 1; Fig. 3). This southward shift is observed to be a further 127.47 km by the 2070s (Table 1, Fig. 3).

**Table 1:** Summary of climate change scenarios on *Ctenocephalides felis* in Australia

Scenario		Current area lost (km^2^)	Area of expansion from current area (km^2^)	% change in area	Area common to current (km^2^)	% current distribution retained	Distance to current centroid (km)	Rate of habitat change (km/decade)
2050: All	RCP2.6	301,018.50	22,108.26	29.08	658,022.32	68.61	242.25	75.70
	RCP4.5	381,786.81	17,326.07	38.00	577,255.01	60.19	271.92	84.98
	RCP6.0	331,719.37	33,929.15	31.05	627,322.45	65.41	338.55	105.80
	RCP8.5	436,877.55	26,587.09	42.78	522,164.27	54.45	397.14	124.11
2050: Clade ‘Sydney’	RCP2.6	339,388.78	21,636.39	29.70	730,538.19	68.28	215.18	67.24
	RCP4.5	439,006.97	11,379.71	39.97	630,919.96	65.79	264.77	82.74
	RCP6.0	383,161.11	26,691.03	33.32	686,765.86	71.61	295.67	92.40
	RCP8.5	501,527.03	21,790.32	44.84	568,399.94	59.27	391.65	122.39
2050: Clade ‘Darwin’	RCP2.6	55,972.30	24,097.10	18.77	113,808.92	67.03	93.97	29.37
	RCP4.5	29,695.90	45,457.57	9.28	140,085.23	82.93	157.61	49.25
	RCP6.0	63,586.33	17,515.69	27.14	106,194.89	62.55	56.51	17.70
	RCP8.5	40,364.56	51,701.02	6.68	129,416.66	76.23	128.77	40.24
2050: Clade ‘Cairns’	RCP2.6	47,923.57	16,947.30	31.07	51,779.46	51.93	206.82	64.50
	RCP4.5	28,949.99	34,684.10	5.75	70,753.04	70.96	252.40	78.88
	RCP6.0	49,881.97	17,574.75	32.40	49,820.06	49.97	132.11	41.28
	RCP8.5	49,642.17	29,042.64	20.66	50,060.86	50,21	312.68	97.71
2070: All	RCP2.6	280,744.69	38,814.52	25.23	678,297.13	70.72	232.17	44.65
	RCP4.5	426,441.54	18,276.39	42.56	532,600.28	55.53	343.84	66.12
	RCP6.0	478,536.97	15,717.72	48.26	480,504.85	50.10	353.93	68.06
	RCP8.5	589,158.53	16,349.90	59.73	369,883.29	38.57	524.61	100.89
2070: Clade ‘Sydney’	RCP2.6	318,234.54	36,549.23	26.33	751,692.43	70.26	218.69	42.06
	RCP4.5	495,955.62	12,628.19	45.17	573,971.35	53.65	332.05	63.86
	RCP6.0	526,910.57	10,408.90	48.27	507,016.40	47.39	347.37	71.99
	RCP8.5	672,242.71	9979.55	61.90	297,684.26	27.82	521.00	100.19
2070: Clade ‘Darwin’	RCP2.6	30,060.54	70,319.91	23.71	139,720.68	82.30	132.10	25.40
	RCP4.5	52,684.02	24,798.35	16.42	117,097.20	68.70	131.59	25.31
	RCP6.0	50,754.84	41,335.67	55.48	119,026.38	70.11	151.47	29.13
	RCP8.5	51,142.07	17,440.52	19.85	118,639.16	69.88	169.62	32.62
2070: Clade ‘Cairns’	RCP2.6	25,371.50	45,879.01	20.57	74,331.53	74.55	224.40	43.15
	RCP4.5	43,708.87	8466.85	35.35	55,994.16	56.16	188.22	36.20
	RCP6.0	49,885.82	11,535.67	38.46	49,817.21	49.97	186.34	35.83
	RCP8.5	56,555.56	6707.94	50.00	43,147.47	43.28	193.32	37.18

*Note*: Total area predicted to be occupied at > 50% probability, distance from each projected centroid and rate per decade of habitat change

**Fig. 4:**
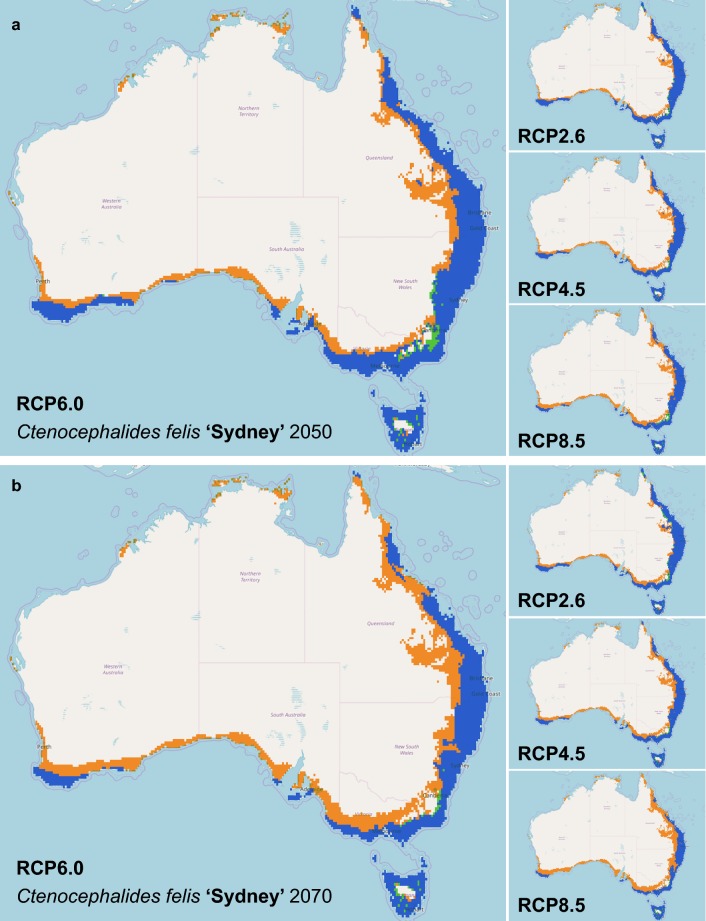
Comparison of climate change models of *Ctenocephalides felis* Clade ‘Sydney’ for 2050 and 2070 in Australia. In 2050 (**a**) and 2070 (**b**), Clade ‘Sydney’ is predicted to reduce its presence in all scenarios. Overall, Clade ‘Sydney’ is predicted to be restricted even closer to the coastline as the inner regions become too warm and dry for flea survival. There is a small expansion predicted for the Blue Mountains range west of Sydney, New South Wales. Orange areas indicate a contraction from the current range, blue areas indicate no change in range and green areas indicate an expansion of the current range. The species distribution models were analysed further using a Maxent climate change model for predicted future distribution of *C. felis* Clade ‘Sydney’ in Australia. IPCC Representative Concentration Pathways (RCP) (2.6, 4.5, 6.0, and 8.5) for the 2050 (**a**) and 2070 (**b**) were evaluated with WorldClim data and 10 arcmin resolution

**Fig. 5:**
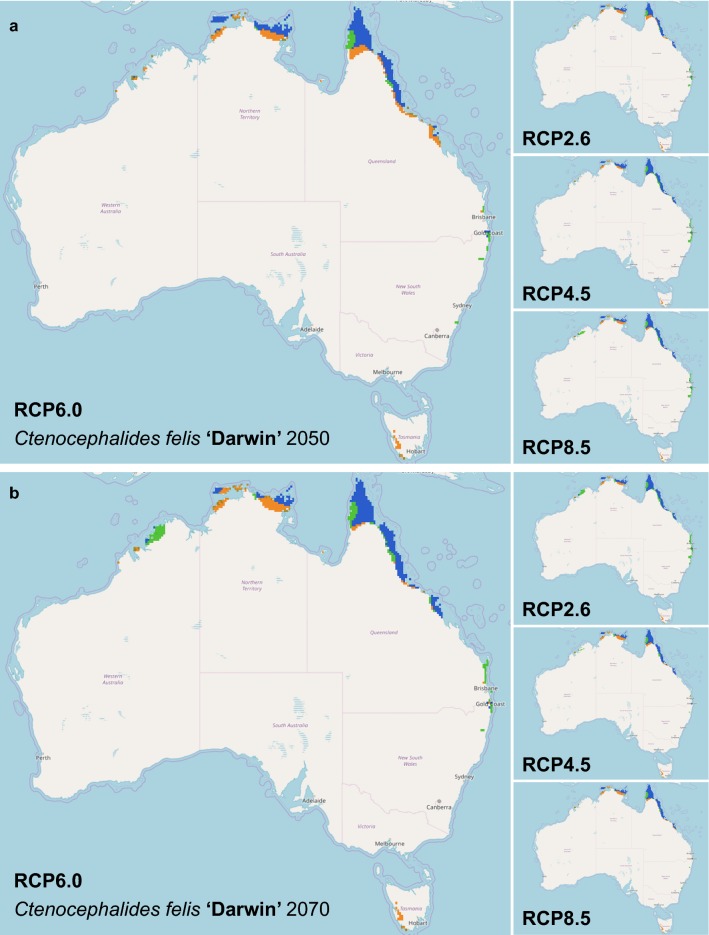
Comparison of climate change models of *Ctenocephalides felis* Clade ‘Darwin’ for 2050 and 2070 in Australia. In 2050 (**a**) and 2070 (**b**), Clade ‘Darwin’ is predicted to reduce its presence in the northern parts of their range and expand its range to further southern Queensland as the northern tropical regions become too warm and dry for flea survival in Australia. Orange areas indicate a contraction from the current range, blue areas indicate no change in range and green areas indicate an expansion of the current range. The species distribution models were analysed further using a Maxent climate change model for predicted future distribution of *C. felis* Clade ‘Darwin’ in Australia. IPCC Representative Concentration Pathways (RCP) (2.6, 4.5, 6.0, and 8.5) for the 2050 (**a**) and 2070 (**b**) were evaluated with WorldClim data and 10 arcmin resolution

**Fig. 6:**
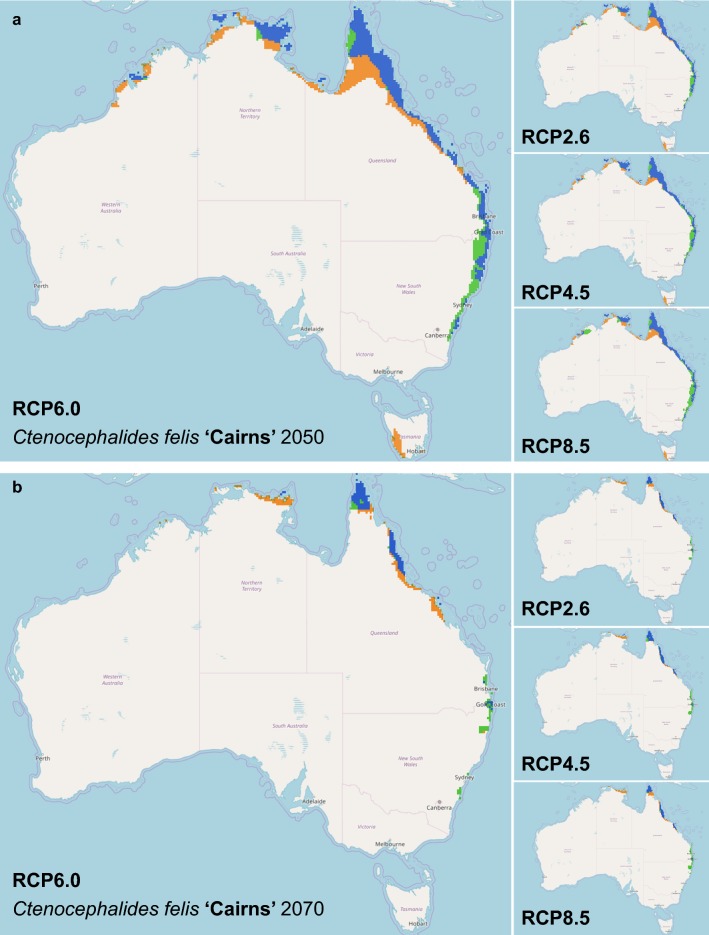
Comparison of climate change models of *Ctenocephalides felis* Clade ‘Cairnsʼ for 2050 and 2070 in Australia. In 2050 (**a**) and 2070 (**b**), Clade ‘Cairns’ is predicted to be further restricted to the eastern coastline of Australia as the inner regions become too warm and dry for flea survival and predicted to make their way south along the east coast. Orange areas indicate a contraction from the current range, blue areas indicate no change in range and green areas indicate an expansion of the current range. The species distribution models were analysed further using a Maxent climate change model for predicted future distribution of *C. felis* Clade ‘Cairns’ in Australia. IPCC Representative Concentration Pathways (RCP) (2.6, 4.5, 6.0, and 8.5) for the 2050 (**a**) and 2070 (**b**) were evaluated with WorldClim data and 10 arcmin resolution

Similar results were seen amongst the three individual clades where the area of suitable climatic conditions for survival of *C. felis* haplotypes declined by 2050 and further by 2070. In 2050 and 2070 respectively, 590,190.26–752,174.58 km^2^ and 307,663.81–788,241.66 km^2^ out of the current 1,069,926.97 km^2^ area is suitable for Clade ‘Sydney’ haplogroup (Table 1). The predicted distribution has a similarity of 59.27–71.61% and 27.83–70.26% in 2050 and 2070, respectively, to the current model (Table 1, Fig. 4). This resulted in a subsequent shift rate of 122.11 km south per decade, with loss of the current northern range (Table 1, Fig. 4). Clade ‘Darwin’ is predicted to experience loss in suitable area, where 123,710.58–185,542.80 km^2^ and 141,895.55–210,040.59 km^2^ in 2050 and 2070 respectively, out of the current 169,781.22 km^2^ is suitable (Table 1). The predicted distribution has a similarity of 62.55–82.93% and 68.70–82.30% in 2050 and 2070, respectively, to the current suitable area for *C. felis* (Table 1, Fig. 5). This resulted in a subsequent shift rate of 49.25 km south per decade (Table 1, Fig. 5). Clade ‘Cairns’ has similarly observed a loss in suitable area, with a loss of 67,394.81–105,437.14 km^2^ and 49,855.41–120,210.54 km^2^ of the current 99,703.03 km^2^ range in 2050 and 2070, respectively (Table 1). The predicted distribution has a similarity of 49.97–70.96% and 43.28–74.55% in 2050 and 2070, respectively, to the current suitable area for *C. felis* (Table 1, Fig. 6). This resulted in a southward range shift of 97.71 km per decade for Clade ‘Cairns’, with loss of the current northern range (Table 1, Fig. 6).

## Discussion

This study has doubled the number of fleas genotyped in Australia and provided an updated perspective on the diversity of *C. felis*, particularly in the north-eastern region. Only three *cox*1 haplotypes have previously been reported from 47 *C. felis* specimens collected from domestic cats and dogs across Australia [1, 2, 5, 7]. This study confirms the presence of the haplotypes (h1, h4 and h5) previously genotyped, and uncovers the existence of three new *cox*1 haplotypes (h2, h3 and h6) in north-eastern Australia. These results provide strong evidence supporting intra-specific diversification of *C. felis* between the north-eastern region and middle to southern regions of Australia. As haplotypes h4, h5 and h6 have only been detected in this tropical region and differ genetically by one SNP, they have been categorised as Clade ‘Cairns’, whilst genetically similar haplotypes h1 and h2 have been found elsewhere have been grouped as Clade ‘Sydney’.

The findings from this study suggest a range expansion of tropical haplogroups as only Clade ‘Sydney’ haplogroup was previously observed in the surrounding area of Cairns in 2014 [1, 2]. However, the theory of emerging haplotypes is questionable as the one dog that was sampled in the outer area of Cairns may not have presented the emerging haplotypes [1, 2]. It was unexpected to observe haplotype h3 in the Cairns region as previously it has only been found in Galiwinku, near Darwin (our unpublished data). Clade ‘Darwin’ may have been present throughout the northern tropical regions before this study and not sampled as only 25 samples have been previously genotyped in these regions.

An initial bottleneck restricting gene flow of the flea populations into Australia could have been the cause of the low haplotype diversity currently present [24]. Over time, the result of extensive transportation and the movement of cats and dogs has facilitated interaction between flea populations [25]. Additionally, the abundance of feral cats and dogs could expedite greater genetic transfer between flea populations [25]. Despite an increase in diversity suggested by this study, it still reveals relatively low mitochondrial variability and highly restricted haplogroup populations of *C. felis* in Australia. The finding of six mtDNA haplotypes is considered to be unusually low compared to other flea species, such as the ten haplotypes of *Pulex simulans* and 24 haplotypes of *Oropsylla hirsuta*, the prairie dog flea [26, 27]. It is expected that for at least every ten samples of mtDNA sequences greater than 500 bp (e.g. *cox*1), multiple haplotypes are expected for one species of animal [28]. Nevertheless, low mitochondrial variability has also been observed in other arthropod species [29]. High homology was similarly found at the *cox*1 and *cox*2 mtDNA regions in a study of *Anopheles stephensi stephensi*, an important malarial vector in India [29]. Oshaghi et al. [29] suggested the low genetic variation is a result of unrestricted gene flow due to the lack of geographical barriers. This, along with extensive movement of hosts, could be the potential contributing factor for the low genetic variation found within Australia [30].

The extensive movement of host allows indirect dispersal of fleas over a large range; however, the results from this study and two previous studies [1, 2] suggest three genetically distinct haplogroups of *C. felis* in Australia. These genetically distinct groups indicate a lack of gene flow between some populations [31]. The species diversification is likely to be influenced by climatic variation as climatic differences can impede gene flow despite sufficient opportunity for dispersal from frequent movement of hosts [4].

The model for the present distribution of *C. felis* in Australia shows that the most suitable habitat is located along the coastline as it presents the ideal environment for flea survival. The environmental conditions of the eastern coast of Australia consists of moderate to warm temperatures and high levels of precipitation that are within the critical thresholds for *C. felis* survival (Australian Bureau of Meteorology, Australian Government; http://www.bom.gov.au/iwk/climate_zones/map_1.shtml) [17, 18]. The Clade ‘Darwin’ model revealed that the range of haplotype h3 is along the northern coastal environments. This model for Clade ‘Darwin’ requires additional samples from these remote and scarcely populated areas to be included to further refine the model. *Ctenocephalides felis* does not have the ability survive and develop in temperatures greater than 35 °C as is seen in inland regions [32].

The predictive models indicate that there is a tropical ecological niche for Clade ‘Cairns’ and Clade ‘Darwin’, whereas Clade ‘Sydney’ has the ability to inhabit most Australian climates. Species diversification as a result of climate as a natural barrier has been observed in other species. The climatic variation between the northern and southern part of Fukushima and Ibaraki prefectures in Japan, with mean temperatures of 18.3 °C and 17 °C respectively, is thought to be the reason for the presence of two genetically distinct groups of the sorghum plant bug, *Stenotus rubrovittatus* [33]. Australia has a very variable climate across the continent where the northern region presents a tropical climate that transitions into a temperate climate in the southern states (Australian Bureau of Meteorology, Australian Government; http://www.bom.gov.au/). Fleas dispersed from host movement may not survive, reproduce or parasitise hosts outside their origin due to movement to a new unfavourable habitat [34].

As climate is a fundamental driver in shaping species diversification, climate change can lead to a range shift or reduction in habitat suitability [35]. Under all RCP climatic scenarios, a southward shift in the distribution of *C. felis* was observed to occur along the coastal areas of Australia by the 2050s and continuing further by the 2070s (range shift = 124.11 km per decade). Although the proximate expansion of *C. felis* into new suitable regions continues by extensive movement of their hosts, our findings predict an ultimate decline of 61.43% in suitable climatic habitat by the 2070s under the RCP8.5 model. The decrease in suitable habitat further north is most likely due to a predicted increase in the maximum temperature of the warmest month. An increase in temperature can increase the rate of development for arthropods; however, the positive association between temperature and developmental rate may be offset by the species total bioclimatic requirement for survival [36]. Initially, the increased temperatures may promote the spread and occurrence of *C. felis* along the north-eastern coastline, but the expected temperature increase of 1.1–6.4 °C from 1980 to 2099 will exceed critical threshold temperatures (35 °C) for *C. felis* survival in the current northern areas of habitat [37]. As a result, the climatic requirements for the growth and development of *C. felis* will become limited in these areas and consequently restrict the distribution of the parasite to areas further south with sufficient bioclimatic resources [37]. Climate change in the future is also expected to increase precipitation and humidity levels in some areas while other areas will experience severe drought conditions [38]. It is known that flea infestations are mostly absent in the Australian inland communities as drought conditions are too harsh for flea survival [39]. As *C. felis* development and survival is highly dependent on moist environments, distribution is most likely to be found in those regions [17]. As moist regions are becoming further restricted to coastlines in the future, the model suggests that there will be an increase in flea populations in these areas.

## Conclusions

A change in the *status quo* of *C. felis* genetic structure will be seen where tropical haplogroups may out-perform or displace the temperate haplogroups further south. Under the proposed IPCC climate change scenarios, a southward shift of *C. felis* range within Australia will occur. Predictive models for the spread of species have been proven to be beneficial epidemiological tools in disease control programs [9, 40, 41]. To gain a better understanding into intraspecific biological variations and ecology of the haplotypes of *C. felis*, it will be necessary to directly compare the development of genetically defined *C. felis* strains under laboratory conditions with defined bioclimatic variables [42, 43].

## Additional files

**Additional file 1: Table S1.** Summary of *Ctenocephalides felis* specimens collected in Cairns and the surrounding region. **Table S2.** Supplementary dataset of *Ctenocephalides felis* samples collated and used in this study. **Table S3.** Summary of *Ctenocephalides felis,* characterised morphologically and genotyped using the mtDNA *cox*1 region.

**Additional file 2: Table S4.** Longitude/latitude of the included *Ctenocephalides felis* samples.

**Additional file 3: Figure S1.** Bioclimatic response graphs for *Ctenocephalides felis* in Australia.

## Abbreviations

*cox*1: cytochrome *c* oxidase subunit 1 gene
mtDNA: mitochondrial DNA
Maxent: Maximum Entropy
IPCC: Intergovernmental Panel on Climate Change
RCP: Representative Concentration Pathways
*cox*2: cytochrome *c* oxidase subunit 2 gene
PCR: polymerase chain reaction

## References

[1] Šlapeta J, King J, McDonell D, Malik R, Homer D, Hannan P, Emery D (2011). The cat flea (*Ctenocephalides f. felis*) is the dominant flea on domestic dogs and cats in Australian veterinary practices. Vet Parasitol..

[2] Lawrence AL, Brown GK, Peters B, Spielman DS, Morin-Adeline V, Šlapeta J (2014). High phylogenetic diversity of the cat flea (*Ctenocephalides felis*) at two mitochondrial DNA markers. Med Vet Entomol..

[3] Rust KM (2017). The biology and ecology of cat fleas and advancements in their pest management: a review. Insects..

[4] Krasnov BR, Shenbrot GI, Khokhlova IS, Poulin R (2005). Diversification of ectoparasite assemblages and climate: an example with fleas parasitic on small mammals. Global Ecol Biogeogr..

[5] Lawrence AL, Hii SF, Chong R, Webb CE, Traub R, Brown G, Šlapeta J (2015). Evaluation of the bacterial microbiome of two flea species using different DNA-isolation techniques provides insights into flea host ecology. FEMS Microbiol Ecol..

[6] Lawrence AL, Hii S-F, Jirsová D, Panáková L, Ionică AM, Gilchrist K (2015). Integrated morphological and molecular identification of cat fleas (*Ctenocephalides felis*) and dog fleas (*Ctenocephalides canis*) vectoring *Rickettsia felis* in central Europe. Vet Parasitol..

[7] Chandra S, Forsyth M, Lawrence AL, Emery D, Šlapeta J (2017). Cat fleas (*Ctenocephalides felis*) from cats and dogs in New Zealand: molecular characterisation, presence of *Rickettsia felis* and *Bartonella clarridgeiae* and comparison with Australia. Vet Parasitol..

[8] Phillips SJ, Anderson RP, Schapire RE (2006). Maximum entropy modeling of species geographic distributions. Ecol Model..

[9] York EM, Butler CJ, Lord WD (2014). Global decline in suitable habitat for *Angiostrongylus* (=*Parastrongylus*) *cantonensis*: the role of climate change. PLoS One..

[10] Gálvez R, Musella V, Descalzo MA, Montoya A, Checa R, Marino V (2017). Modelling the current distribution and predicted spread of the flea species *Ctenocephalides felis* infesting outdoor dogs in Spain. Parasit Vectors..

[11] Phillips SJ, Dudik M, Schapire RE (2004). A maximum entropy approach to species distribution modeling. Proceedings of the 21st International Conference on Machine Learning.

[12] Escobar LE, Craft ME (2016). Advances and limitations of disease biogeography using ecological niche modeling. Front Microbiol..

[13] Hopkins GHE, Rothschild M. Volume 1: Tungidae and Pulicidae. In: An illustrated catalogue of the Rothschild collection of fleas (Siphonaptera) in the British Museum (Natural History): with keys and short descriptions for the identification of families, genera, species and subspecies, vol. 1. London: The Trustees of the British Museum; 1953.

[14] Dunnet G, Mardon D (1974). A monograph of Australian fleas (Siphonaptera). Aus J Zool Suppl Ser..

[15] Folmer O, Black M, Hoeh W, Lutz R, Vrijenhoek R (1994). DNA primers for amplification of mitochondrial cytochrome c oxidase subunit I from diverse metazoan invertebrates. Mol Mar Biol Biotechnol..

[16] Hallgren W, Beaumont L, Bowness A, Chambers L, Graham E, Holewa H (2016). The biodiversity and climate change virtual laboratory: where ecology meets big data. Environ Model Softw.

[17] Silverman J, Rust MK (1983). Some abiotic factors affecting the survival of the cat flea, *Ctenocephalides felis* (Siphonaptera: Pulicidae). Environ Entomol..

[18] Dryden MW (1989). Host association, on-host longevity and egg production of *Ctenocephalides felis* felis. Vet Parasitol..

[19] Lobo Jorge M, Jiménez-Valverde A, Real R (2007). AUC: a misleading measure of the performance of predictive distribution models. Global Ecol Biogeogr..

[20] van Vuuren DP, Stehfest E, den Elzen MGJ, Kram T, van Vliet J, Deetman S (2011). RCP2.6: exploring the possibility to keep global mean temperature increase below 2 °C. Clim Change..

[21] Thomson AM, Calvin KV, Smith SJ, Kyle GP, Volke A, Patel P (2011). RCP4.5: a pathway for stabilization of radiative forcing by 2100. Clim Change..

[22] Masui T, Matsumoto K, Hijioka Y, Kinoshita T, Nozawa T, Ishiwatari S (2011). An emission pathway for stabilization at 6 Wm^−2^ radiative forcing. Clim Change..

[23] Riahi K, Rao S, Krey V, Cho C, Chirkov V, Fischer G (2011). RCP 85 - a scenario of comparatively high greenhouse gas emissions. Clim Change..

[24] Maron M, McAlpine CA, Watson JEM, Maxwell S, Barnard P (2015). Climate-induced resource bottlenecks exacerbate species vulnerability: a review. Divers Distrib..

[25] Glen AS, Dickman CR (2005). Complex interactions among mammalian carnivores in Australia, and their implications for wildlife management. Biol Rev Camb Philos Soc..

[26] de la Cruz KD, Whiting MF (2003). Genetic and phylogeographic structure of populations of *Pulex simulans* (Siphonaptera) in Peru inferred from two genes (CytB and CoII). Parasitol Res..

[27] Brinkerhoff RJ, Martin AP, Jones RT, Collinge SK (2011). Population genetic structure of the prairie dog flea and plague vector, *Oropsylla hirsuta*. Parasitology..

[28] Avise JC (2000). Phylogeography: the history and formation of species.

[29] Oshaghi MA, Yaaghoobi F, Abaie MR (2006). Pattern of mitochondrial DNA variation between and within *Anopheles stephensi* (Diptera: Culicidae) biological forms suggests extensive gene flow. Acta Trop..

[30] Johnson D (2009). The geology of Australia.

[31] Dryden MW, Rust MK (1994). The cat flea: biology, ecology and control. Vet Parasitol..

[32] Bitam I, Dittmar K, Parola P, Whiting MF, Raoult D (2010). Fleas and flea-borne diseases. Int J Infect Dis..

[33] Kobayashi T, Matsuki N, Yokosuka T (2011). Genetic isolation of the sorghum plant bug *Stenotus rubrovittatus* (Hemiptera: Miridae) in Fukushima and Ibaraki prefectures. Appl Entomol Zool..

[34] Mize EL, Tsao JI, Maurer BA (2011). Habitat correlates with the spatial distribution of ectoparasites on *Peromyscus leucopus* in southern Michigan. J Vector Ecol..

[35] Lafferty KD (2009). The ecology of climate change and infectious diseases. Ecology..

[36] McArthur RH (1972). Geographical ecology: patterns in the distribution of a species.

[37] Houghton JT, Ding Y, Griggs DJ, Noguer M, van der Linden PJ (2001). Climate change 2001: the scientific basis.

[38] Ramirez J, Jarvis A (2008). High resolution statistically downscaled future climate surfaces.

[39] Banks AW (1952). Some animal parasites of the Northern Territory, and some remarks. Aus Vet J..

[40] González C, Wang O, Strutz SE, González-Salazar C, Sánchez-Cordero V, Sarkar S (2010). Climate change and risk of leishmaniasis in North America: predictions from ecological niche models of vector and reservoir species. PLoS Negl Trop Dis..

[41] Peterson AT, Shaw J (2003). *Lutzomyia* vectors for cutaneous leishmaniasis in southern Brazil: ecological niche models, predicted geographic distributions, and climate change effects. Int J Parasitol..

[42] Yao KP, Ngoran KE, Franc M (2006). Étude de quelques paramètres écologiques de *Ctenocephalides felis strongylus* (Jordan, 1925) (Siphonaptera: Pulicidae). Parasite..

[43] Yao KP, NʼGoran KE, Franc M (1925). Influence de la température sur le développement de la puce Africaine du chat *Ctenocephalides felis* strongylus (Jordan, 1925) (Siphonaptera : Pulicidae. Parasite..

