# Supplementary material for: Climate change models predict southerly shift of the cat flea (Ctenocephalides felis) distribution in Australia
Source: Parasit Vectors. 2019 Mar 22;12:137. doi: 10.1186/s13071-019-3399-6 (PMC6431004; doi:10.1186/s13071-019-3399-6)
Supplement: Supplementary file 1 — Additional file 1: Table S1. Summary of Ctenocephalides felis specimens collected in Cairns and the surrounding region. Table S2. Supplementary dataset of Ctenocephalides felis samples collated and used in this study. Table S3. Summary of Ctenocephalides felis, characterised morphologically and genotyped using the mtDNA cox1 region. [file 13071_2019_3399_MOESM1_ESM.docx]

**Additional file 1: Table S1.** Summary of *Ctenocephalides felis* specimens collected in Cairns and the surrounding region.

| Location | Elevation (m) | Latitude | Longitude | Host(s) | Specimens | | | Sequence ID |
| --- | --- | --- | --- | --- | --- | --- | --- | --- |
|  |  |  |  |  | No. | Female | Male |  |
| Aloomba 4871 | 354 | -17.1071 | 145.8317 | 1 cat | 3 | 3 | 0 | JS4350-JS4354 |
| Atherton 4883 | 753.5 | -17.2607 | 145.4772 | 1 cat | 1 | 0 | 1 | JS4234 |
| Bentley Park 4869 | 14.6 | -17.0034 | 145.7422 | 2 dogs | 10 | 5 | 5 | JS4235,  JS4276-JS4277,  JS4526-JS4532 |
| Earlville 4870 | 7.5 | -16.9410 | 145.7407 | 1 dog | 28 | 21 | 7 | JS4278-JS4280,  JS4540-JS4547,  JS4554-JS4570 |
| Gordonvale 4865 | 357 | -17.0832 | 145.7961 | 1 dog | 5 | 4 | 1 | JS4353-JS4354, JS4551-JS4553 |
| Mareeba 4880 | 405.7 | -16.9913 | 145.4235 | 2 dogs | 19 | 16 | 3 | JS4300-JS4305, JS4355-JS4362, JS4340-JS4344 |
| Tanawha 4556 | 28.7 | -26.7129 | 153.0488 | 1 dog | 1 | 1 | 0 | JS4236 |
| Trinity Beach 4879 | 17 | -16.8052 | 145.6902 | 1 dog,  3 cats | 4 | 3 | 1 | JS4345-JS4346,  JS4348-JS4349 |
| Woree 4868 | 20 | -16.9634 | 145.7461 | 1 dog,  3 cats | 10 | 9 | 1 | JS4548-JS4550,  JS4525,  JS4533-JS4538 |

**Note: Out of the 81 *C. felis* collected, 62 were female and 19 were male (Sex ratio 3.263).**

**Additional file 1: Table S2.** Supplementary dataset of *Ctenocephalides felis* samples collated and used in this study.

| Location | Elevation (m) | Latitude | Longitude | Host(s) | Specimens | | Haplotype | Sequence ID | Paper |
| --- | --- | --- | --- | --- | --- | --- | --- | --- | --- |
|  |  |  |  |  | **Female** | **Male** |  |  |  |
| Albion Park 2527 | 21 | -34.5759 | 150.7737 | cow | 0 | 3 | 1 | - | Unpublished |
| Ambarvale 2560 | 90 | -34.0833 | 150.8007 | cat | 1 | 0 | 1 | - | Unpublished |
| Ballarat 3355 | 461 | -37.539731 | 143.823297 | cat | 1 | 0 | 1 | MG586438 | Šlapeta et al., 2011 |
| Bankstown 2200 | 13 | -33.925922 | 151.033758 | dog | 1 | 0 | 1 | KF684894 | Šlapeta et al., 2011 |
| Baulkham Hills 2153 | 101 | -33.7629 | 150.9921 | dog | 7 | 0 | 1 | MG586441; MG586444- MG586449 | Lawrence et al., 2015 |
| Bega 2550 | 27 | -36.666614 | 149.8244 | dog | 0 | 2 | 1 | KF684885.2 | Šlapeta et al., 2011; unpublished |
| Bindoon 6502 | 152 | -31.3800 | 116.0970 | cat | 1 | 0 | 1 | - | Unpublished |
| Blue Mountains 2780 | 999 | -33.717528 | 150.311553 | cat | 1 | 0 | 1 | MG586439 | Šlapeta et al., 2011 |
| Booval 4304 | 25 | -27.610181 | 152.7908 | dog | 0 | 1 | 1 | MG586330 | Šlapeta et al., 2011 |
| Bundaberg 4670 | 11 | -24.855161 | 152.3737 | dog | 0 | 1 | 1 | MG586328 | Šlapeta et al., 2011 |
| Cairns 4870 | 9 | -16.9351 | 145.7452 | dog | 2 | 0 | 4 | - | Unpublished |
| Cairns City 4870 | 3 | -16.925 | 145.775 | dogs/ cats***** | 13 | 4 | 4 | KF684896-KF684912 | Lawrence et al., 2014 |
| Camden 2570 | 81 | 34.0544 | 150.6958 | cat | 0 | 1 | 1 | - | Unpublished |
| Camperdown 2050 | 32 | -33.8901 | 151.1799 | cat | 1 | 0 | 1 | KF684886.1 | Chandra et al., 2017 |
| Cessnock 2325 | 72 | -32.8334 | 151.3540 | dog | 0 | 1 | 1 | HQ696941 | Šlapeta et al., 2011 |
| Coningham 7054 | 73 | -43.0833 | 147.2805 | cat | 0 | 1 | 1 | - | Unpublished |
| Dundas Valley 2117 | 73 | -33.7931 | 151.06 | cat | 1 | 0 | 1 | - | Unpublished |
| East Kurrajong 2758 | 405 | -33.5206 | 150.7191 | cat | 1 | 0 | 1 | KF684882.1 | Lawrence et al., 2014 |
| Eastwood 2122 | 72 | -33.7904 | 151.0817 | cat | 1 | 0 | 1 | - | Unpublished |
| Epping 2121 | 95 | -33.7746 | 151.0788 | cat | 2 | 0 | 1 | MG586451; KF684882.2 | Lawrence et al., 2015 |
| Epping 2121 | 95 | -33.7746 | 151.0788 | dog | 1 | 1 | 1 | - | Unpublished |
| Galiwinku 0822 | 32 | -12.0247 | 135.5685 | dog | 6 | 1 | 3 | - | Unpublished |
| Gawler 5118 | 58 | -34.6069 | 138.7465 | dog | 6 | 0 | 1 | KF684882.2 | Šlapeta et al., 2011; unpublished |
| Glenorie 2157 | 144 | -33.5950 | 150.9710 | cat | 1 | 0 | 1 | KF684883.1 | Lawrence et al., 2014 |
| Hornsby 2077 | 193 | -33.7049 | 151.099 | cat | 2 | 0 | 1 | - | Unpublished |
| Kangaroo Island 5223 | 160 | -35.7752 | 137.2142 | cat | 1 | 0 | 1 | - | Unpublished |
| Kemps Creek 2178 | 48 | -33.8746 | 150.7987 | colony fleas | 1 | 0 | 1 | - | Unpublished |
| Kilsyth 3137 | 122 | -37.803919 | 145.317808 | cat | 1 | 0 | 1 | MG586436 | Šlapeta et al., 2011 |
| Kingston Beach 7050 | 4 | -42.9811 | 147.3228 | cat | 1 | 0 | 1 | - | Unpublished |
| Lara 3212 | 20 | -38.0167 | 144.4167 | cat | 1 | 0 | 1 | - | Unpublished |
| Mareeba 4880 | 408 | -16.993325 | 145.424033 | dog | 0 | 1 | 1 | MG586329 | Šlapeta et al., 2011 |
| Mareeba 4880 | 404 | -17.0019 | 145.4389 | dog | 2 | 1 | 4 | - | Unpublished |
| Minto 2566 | 54 | -34.0254 | 150.849 | cat | 1 | 0 | 1 | - | Unpublished |
| Mogumber 6506 | 198 | -31.0370 | 116.1030 | cat | 0 | 1 | 1 | - | Unpublished |
| Morley 6062 | 25 | -31.902525 | 115.8976 | dog | 2 | 0 | 1 | KF684886.2; MG586450 | Šlapeta et al., 2011; Lawrence et al., 2015 |
| Morwell 3840 | 85 | -38.236764 | 146.4012 | dog | 1 | 0 | 1 | KF684883.2 | Šlapeta et al., 2011 |
| Newcastle 2281 | 12 | -33.0693 | 151.6605 | dog | 1 | 0 | 1 | HQ696942 | Šlapeta et al., 2011 |
| Northbridge 2063 | 93 | -33.8110 | 151.2159 | colony fleas | 1 | 1 | 1 | - | Unpublished |
| Park Ridge 4125 | 47 | -27.689286 | 153.034353 | dog | 0 | 2 | 1 | MG586347; KF684889 | Šlapeta et al., 2011; Lawrence et al., 2014 |
| Port Macquarie 2444 | 7 | -31.4327 | 152.9131 | dog | 0 | 1 | 1 | HQ696943 | Šlapeta et al., 2011 |
| Prospect 2148 | 83 | -33.8029 | 150.9157 | cat | 1 | 0 | 1 | KF684882 | Lawrence et al., 2015 |
| Richmond 3121 | 23 | -42.7333 | 147.4333 | cat | 1 | 0 | 1 | - | Unpublished |
| Rooty hill 2766 | 42 | -33.772728 | 150.844397 | cat | 1 | 0 | 1 | MG586440 | Šlapeta et al., 2011 |
| Ruse 2560 | 105 | -34.065 | 150.8412 | dog | 1 | 0 | 1 | - | Unpublished |
| Seaforth 2092 | 69 | -33.797833 | 151.2504 | cat | 0 | 1 | 1 | MG586365 | Šlapeta et al., 2011 |
| South Hobart 7004 | 56 | -42.8925 | 147.3161 | cat | 1 | 0 | 1 | - | Unpublished |
| Stanmore 2048 | 30 | -33.8942 | 151.1644 | dog | 1 | 1 | 1 | KF684885.1 | Lawrence et al., 2014; unpublished |
| Sydney 2000 | 35 | -33.8945 | 151.1637 | dog | 1 | 0 | 1 | KF684884.1 | Lawrence et al., 2014 |
| Wollongong 2525 | 54 | -34.431875 | 150.8434 | dog | 1 | 0 | 1 | KF684884.2 | Šlapeta et al., 2011 |
| Yarrabah 4871 | 354 | -16.9094 | 145.8703 | 3 dogs, 1 cat | 2 | 1 | 4 | KT376437-KT376439 | Lawrence et al., 2015 |

***fleas were collected by flea traps set up in seventeen different households.**

**Additional file 1: Table S3.** Summary of *Ctenocephalides felis,* characterised morphologically and genotyped using the mtDNA *cox1* region.

| Location | No. specimens | Sequence ID | *Cox1* haplotype | Clade |
| --- | --- | --- | --- | --- |
| Aloomba 4871 | 3 females  0 males | JS4350-JS4352 | h4 | Cairns |
| Atherton 4883 | 0 females  1 male | JS4234* | h4 | Cairns |
| Bentley Park 4869 | 0 females  1 male | JS4276 | h1 | Sydney |
| Bentley Park 4869 | 1 female  0 males | JS4277* | h2 | Sydney |
| Bentley Park 4869 | 3 females  4 males | JS4526-JS4532 | h4 | Cairns |
| Bentley Park 4869 | 1 female  0 males | JS4235* | h5 | Cairns |
| Earlville 4870 | 19 females  7 males | JS4278-JS4280, JS4540-JS4547, JS4554-JS4559,  JS4561,  JS4562, JS4564-JS4570 | h4 | Cairns |
| Earlville 4870 | 2 females  0 males | JS4560*, JS4563 | h6 | Cairns |
| Gordonvale 4865 | 1 female  0 males | JS4354 | h1 | Sydney |
| Gordonvale 4865 | 1 female  0 males | JS4353 | h3 | Darwin |
| Gordonvale 4865 | 2 females  1 male | JS4551-JS4553 | h4 | Cairns |
| Mareeba 4880 | 10 females  1 male | JS4300, JS4303, JS4304, JS4359, JS4361, JS4362 JS4340-JS4344 | h1 | Sydney |
| Mareeba 4880 | 1 female  2 males | JS4356, JS4358, JS4360 | h2 | Sydney |
| Mareeba 4880 | 4 females  0 males | JS4301*, JS4302, JS4305, JS4357 | h3 | Darwin |
| Mareeba 4880 | 1 female  0 males | JS4355 | h4 | Cairns |
| Tanawha 4556 | 1 female  0 males | JS4236* | h1 | Sydney |
| Trinity Beach 4879 | 3 females  1 male | JS4345-JS4346, JS4348-JS4349 | h4 | Cairns |
| Woree 4868 | 9 females  1 male | JS4548-JS4550, JS4525, JS4533-JS4538 | h4 | Cairns |

***reference haplotypes sequences**
