# Supplementary material for: Climate change models predict southerly shift of the cat flea (Ctenocephalides felis) distribution in Australia
Source: Parasit Vectors. 2019 Mar 22;12:137. doi: 10.1186/s13071-019-3399-6 (PMC6431004; doi:10.1186/s13071-019-3399-6)
Supplement: Supplementary file 3 — Additional file 3: Figure S1. Bioclimatic response graphs for Ctenocephalides felis in Australia. [file 13071_2019_3399_MOESM3_ESM.pptx]

## Slide 1
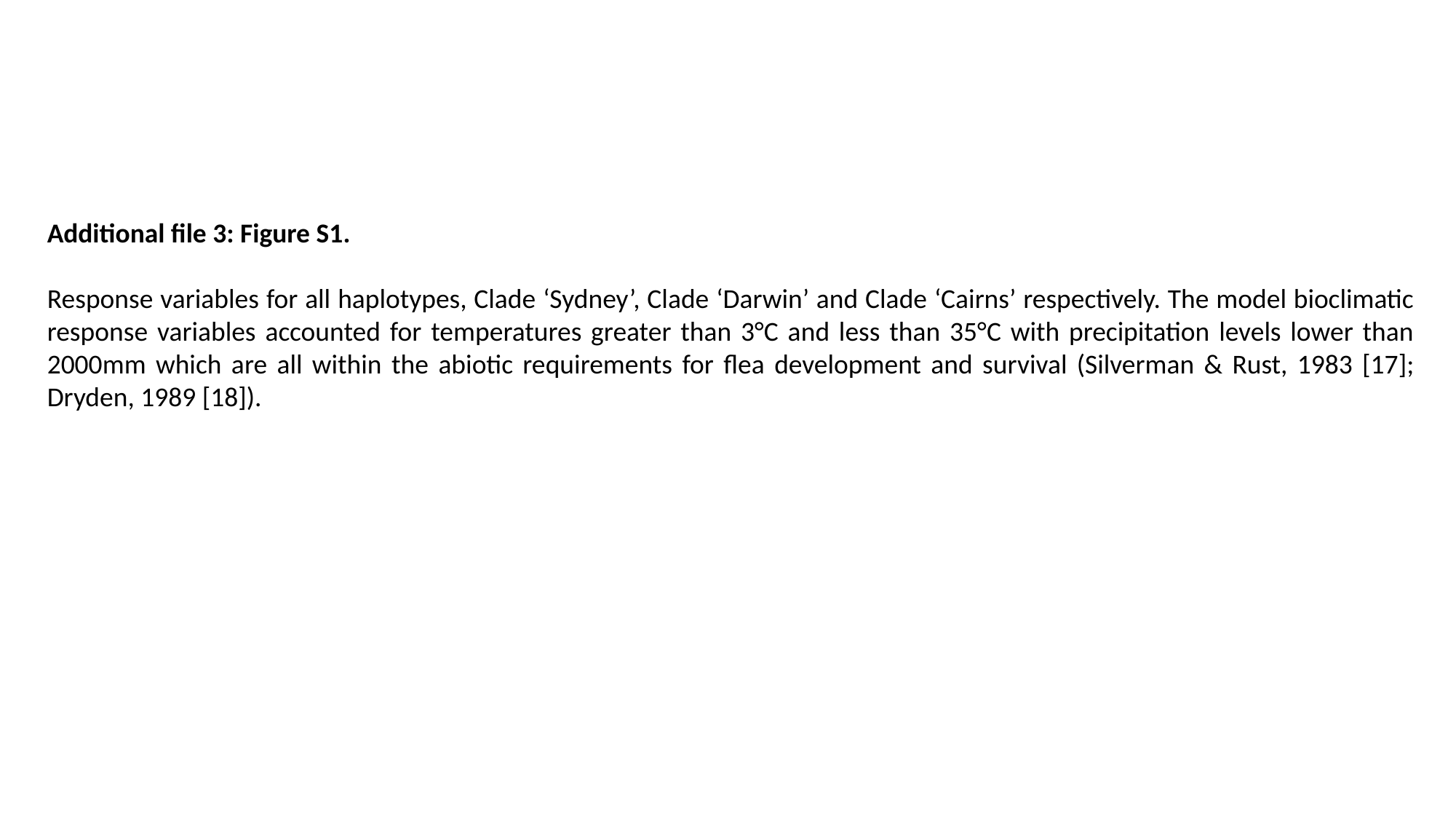

Additional file 3: Figure S1.
Response variables for all haplotypes, Clade ‘Sydney’, Clade ‘Darwin’ and Clade ‘Cairns’ respectively. The model bioclimatic response variables accounted for temperatures greater than 3°C and less than 35°C with precipitation levels lower than 2000mm which are all within the abiotic requirements for flea development and survival (Silverman & Rust, 1983 [17]; Dryden, 1989 [18]).

## Slide 2
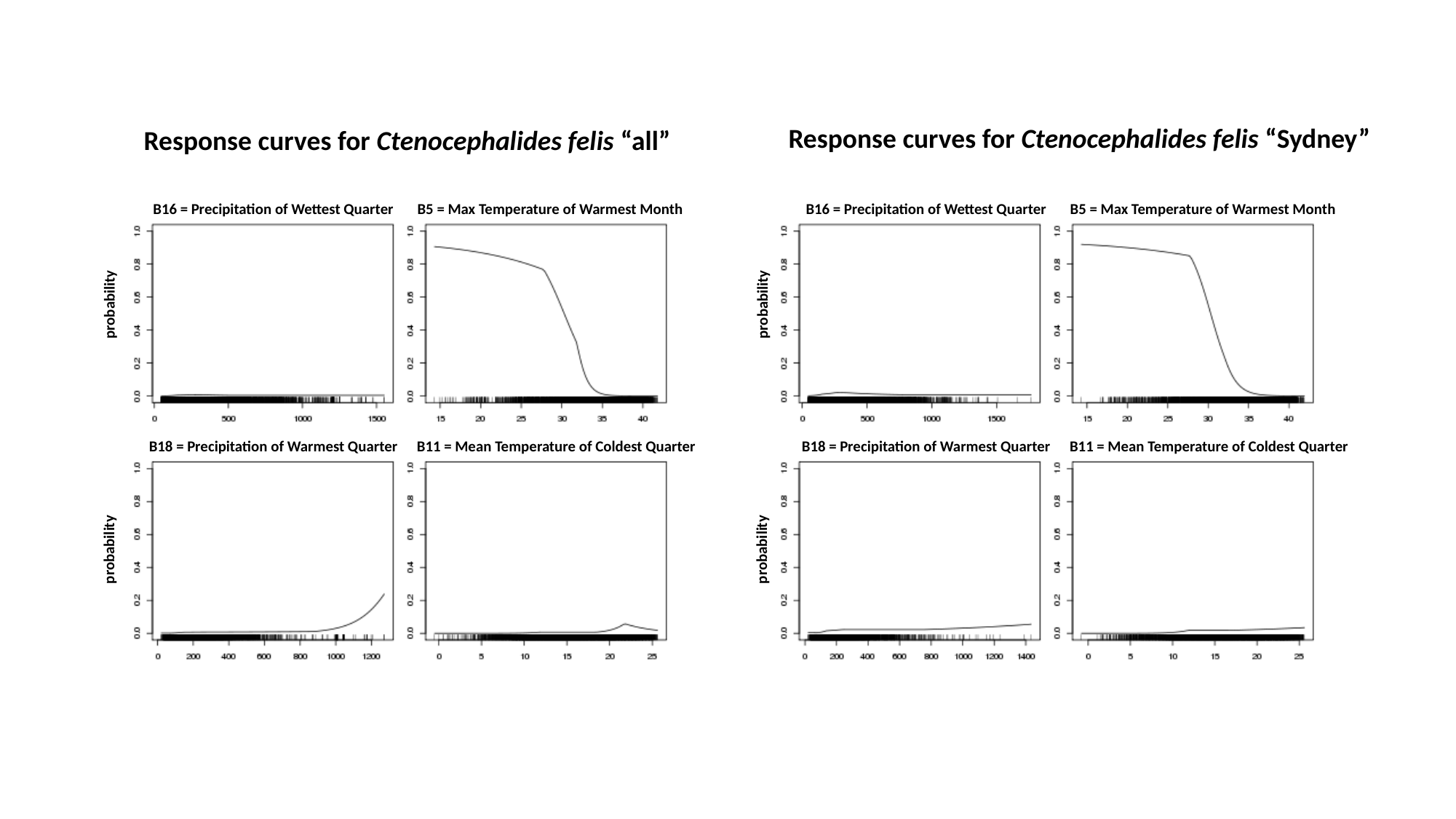

Response curves for Ctenocephalides felis “Sydney”
Response curves for Ctenocephalides felis “all”
B5 = Max Temperature of Warmest Month
B5 = Max Temperature of Warmest Month
B16 = Precipitation of Wettest Quarter
B16 = Precipitation of Wettest Quarter
probability
probability
B18 = Precipitation of Warmest Quarter
B18 = Precipitation of Warmest Quarter
B11 = Mean Temperature of Coldest Quarter
B11 = Mean Temperature of Coldest Quarter
probability
probability

## Slide 3
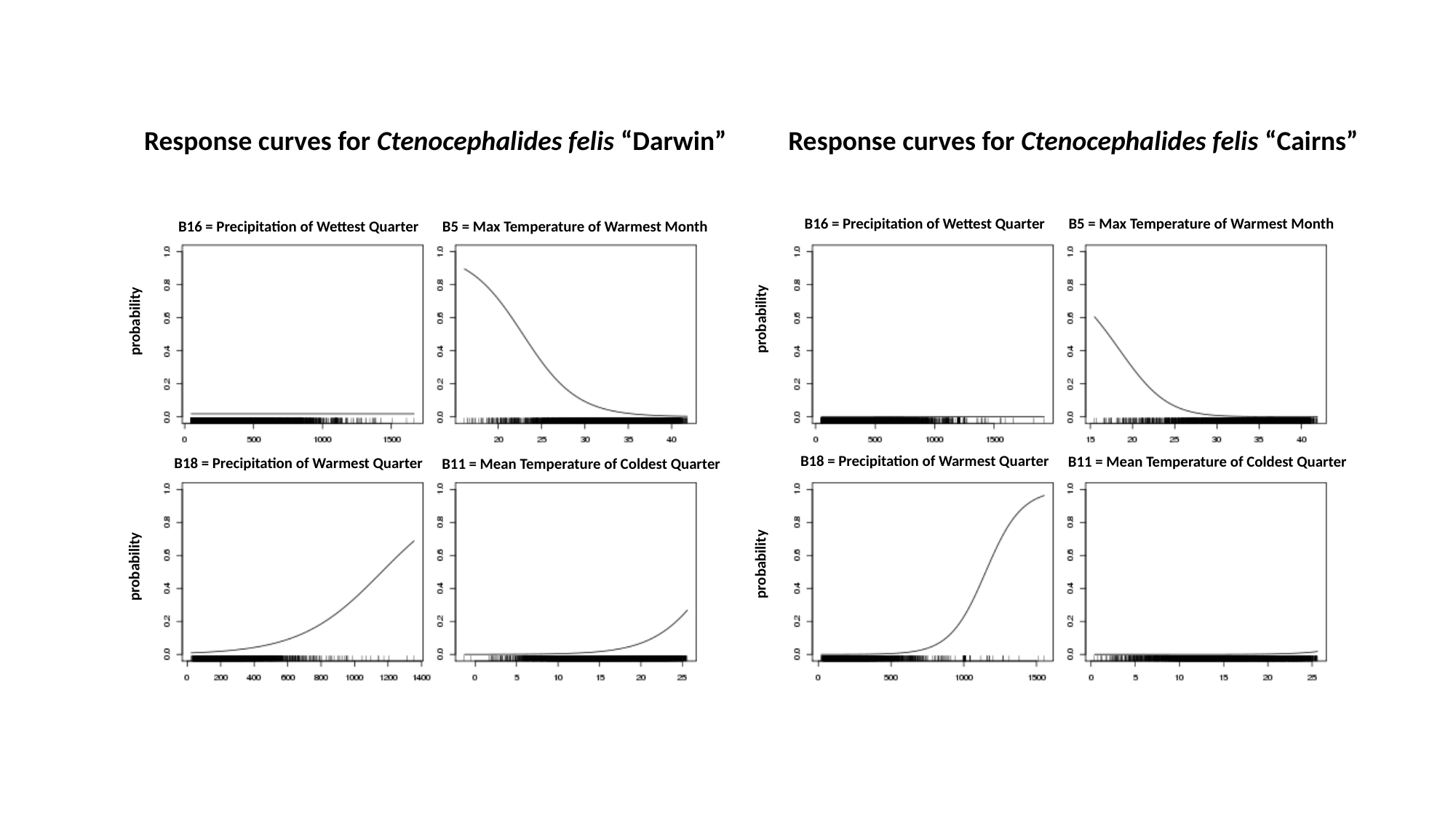

Response curves for Ctenocephalides felis “Cairns”
Response curves for Ctenocephalides felis “Darwin”
B5 = Max Temperature of Warmest Month
B16 = Precipitation of Wettest Quarter
B5 = Max Temperature of Warmest Month
B16 = Precipitation of Wettest Quarter
probability
probability
B18 = Precipitation of Warmest Quarter
B11 = Mean Temperature of Coldest Quarter
B18 = Precipitation of Warmest Quarter
B11 = Mean Temperature of Coldest Quarter
probability
probability
